# Synthesis and *in Vitro* Antiproliferative Evaluation of C-13 Epimers of Triazolyl-d-Secoestrone Alcohols: The First Potent 13α-d-Secoestrone Derivative

**DOI:** 10.3390/molecules21050611

**Published:** 2016-05-12

**Authors:** Johanna Szabó, Nóra Jerkovics, Gyula Schneider, János Wölfling, Noémi Bózsity, Renáta Minorics, István Zupkó, Erzsébet Mernyák

**Affiliations:** 1 Department of Organic Chemistry, University of Szeged, Dóm tér 8, Szeged H-6720, Hungary; johanna.szab@gmail.com (J.S.); jerkovicsnora@gmail.com (N.J.); schneider@chem.u-szeged.hu (G.S.); wolfling@chem.u-szeged.hu (J.W.); 2 Department of Pharmacodynamics and Biopharmacy, University of Szeged, Eötvös u. 6, Szeged H-6720, Hungary; bozsity.noemi@pharm.u-szeged.hu (N.B.); minorics@pharm.u-szeged.hu (R.M.); zupko@pharm.u-szeged.hu (I.Z.)

**Keywords:** antiproliferative effect, azide-alkyne cycloaddition, d-secoestrone, triazole

## Abstract

The syntheses of C-13 epimeric 3-[(1-benzyl-1,2,3-triazol-4-yl)methoxy]-d-secoestrones are reported. Triazoles were prepared from 3-(prop-2-inyloxy)-d-secoalcohols and *p*-substituted benzyl azides via Cu(I)-catalyzed azide-alkyne cycloaddition (CuAAC). The antiproliferative activities of the products and their precursors were determined *in vitro* against a panel of human adherent cervical (HeLa, SiHa and C33A), breast (MCF-7, MDA-MB-231, MDA-MB-361 and T47D) and ovarian (A2780) cell lines by means of MTT assays. The orientation of the angular methyl group and the substitution pattern of the benzyl group of the azide greatly influenced the cell growth-inhibitory potential of the compounds. The 13β derivatives generally proved to be more potent than their 13α counterparts. Introduction of a benzyltriazolylmethyl group onto the 3-OH position seemed to be advantageous. One 13α compound containing an unsubstituted benzyltriazolyl function displayed outstanding antiproliferative activities against three cell lines.

## 1. Introduction

**Figure 1:**
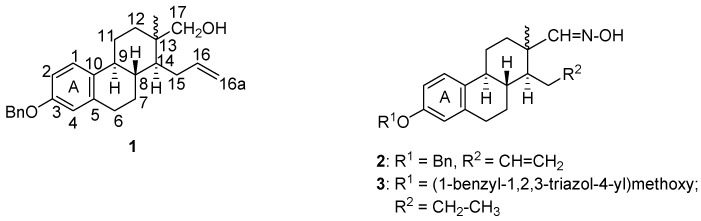
Structures of the potent antiproliferative d-secoestrones **1** and **2**, **3**.

Anticancer drug design based on synthetic modifications of naturally occurring biomolecules may lead to nontoxic drug candidates with selective antitumoral potencies [1,2]. Estrone-based anticancer agents are already utilized in therapy, but one of the most important requirements of these drugs is a lack of original hormonal activity [3,4]. The literature provides evidence that inversion of the configuration at C-13 or the opening of ring D of the estrane core may lead to the loss of estrogenic activity [5,6,7,8,9]. We recently reported that 3-benzyl ethers of d-secoestrone alcohol or oxime (compounds **1** and **2**, Figure 1.) exert substantial *in vitro* cell growth-inhibitory action against a number of cancer cell lines, with IC_50_ values in the low micromolar or submicromolar range [10,11]. Compounds **1** and **2** were diversified at several sites in the molecule, including different modifications (etherifications, esterifications or debenzylations) at C-3 and/or C-17 and epimerization at C-13 (only in the case of **2**). It was concluded that the nature of the 3- and 17-functional groups exerts a great impact on the antiproliferative behavior of the compounds. 3-Ethers proved to be more potent than their 3-OH counterparts. Derivatives containing a 17-oxime function displayed more pronounced cytostatic properties than those of 17-hydroxymethyl derivatives. 3-Hydroxy-d-secooxime **2**, but not the d-secoalcohol **1**, was further derivatized by introducing a terminal alkyne function onto the 3-OH group by using propargyl bromide, and the resulting steroid alkyne was subjected to Cu(I)-catalyzed azide-alkyne cycloaddition (CuAAC) with different substituted benzyl azides. The triazole moiety is frequently used as a linker in bioconjugates, because it is a good mimic of peptide bonding with high proteolytic and metabolic stability [12,13,14]. Introduction of a triazolyl function into the oxime **2** led to improved antiproliferative properties as compared with the 3-benzyl ether. The benzyltriazolylmethoxy d-secooxime **3** (Figure 1) exerted substantial cell growth-inhibitory effects against several human cancer cell lines.

The abovementioned outstanding results suggested that it might be useful to introduce the benzyltriazolyl function onto other positions of the d-secoestrone scaffold. In a continuation of our earlier work, we focused herein on the synthesis of steroidal alkynes bearing a terminal alkyne function on the opened ring D. The d-secoestrone 17-carboxylic acids were reacted with propargylamine using peptide coupling reagents [15]. The reactions were carried out in both the 13α- and the 13β-estrone series in order to obtain more compounds for structure-activity determinations. The resulting *N*-propargyl 17-carboxamides were reacted with small molecule azides, such as substituted benzyl azides, and the resulting triazoles were evaluated for their antiproliferative activities against several human reproductive cancer cell lines. The activities of the compounds depended greatly on the substitution pattern of the aromatic ring of the benzyl azide moiety. 3-Benzyl ethers of 13β-(*p*-alkylbenzyl)triazoles displayed outstanding selective antiproliferative potential against A2780 cells.

There are already a number of literature examples of the synthesis of antiproliferative steroidal triazoles formed mainly from steroid azides and small molecule alkynes, such as phenylacetylenes. It has been found that the cytostatic activity of the resulting triazoles depends considerably on the substitution pattern of the phenyl group of the acetylene [16,17,18,19,20,21,22].

In view of the abovementioned recent results, we aimed to introduce the benzyltriazolylmethoxy moiety onto the 3-OH group of the C-13 epimeric d-secoalcohols. The synthesis of the d-secoestrone alkynes were first planned at position C-3, followed by the CuAAC reactions of the secosteroidal alkynes with substituted benzyl azides. The next goal was to perform comparative investigations of the *in vitro* antiproliferative activities of the products and their precursors by means of MTT assays against a panel of human adherent cervical (HeLa, SiHa and C33A), breast (MCF-7, MDA-MB-231, MDA-MB-361 and T47D) and ovarian (A2780) cell lines. Our main objective was to establish some structure-activity relationship, focusing particularly on the C-13 epimeric character of the compounds.

## 2. Results

**Scheme 1:**
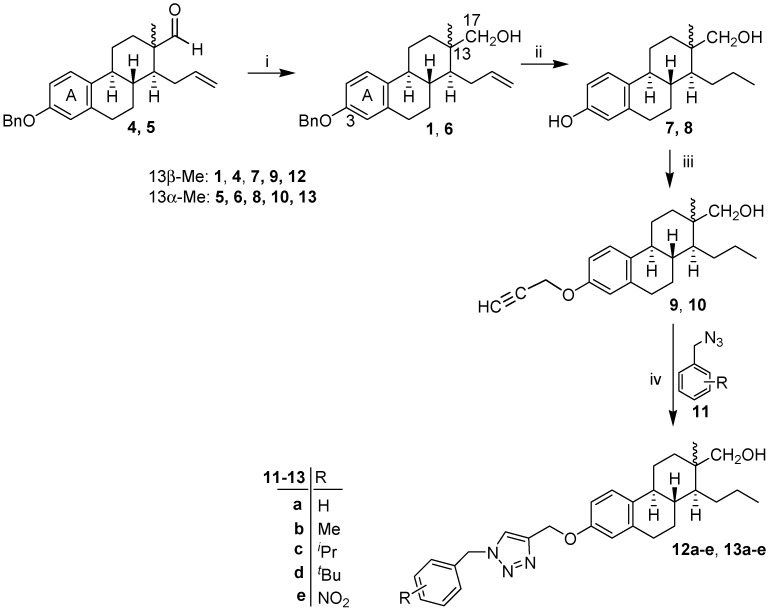
Synthesis of d-secoestrone derivatives (**12a**–**e** and **13a**–**e**). *Reagents and conditions*: (**i**) 5 equiv. KBH_4_, CH_2_Cl_2_:MeOH = 1:1, 0 °C—r.t., 0.5 h; (**ii**) H_2_ (20 bar), Pd/C, EtOAc, 3 h; (**iii**) propargyl bromide (1.5 equiv.), K_2_CO_3_ (7 equiv.), acetone, 70 °C, 24 h; (iv) CuI (0.05 equiv.), Ph_3_P (0.1 equiv.), DIPEA (3 equiv.), toluene, reflux, 2 h.

We first synthesized steroidal alkynes in both the 13α- and the 13β-d-secoestrone series (Scheme 1). After removal of the benzyl protecting group of compounds **1** or **6** by hydrogenolysis, an excess of propargyl bromide was added in the presence of K_2_CO_3_, leading to the 3-(prop-2-inyloxy)-17-alcohols **9** or **10**. The terminal alkynes **9** or **10** were reacted with benzyl azide (**11a**) or its substituted derivatives **11b**–**e** under recently published CuAAC reaction conditions [20], furnishing the desired triazolyl compounds **12a**–**e** or **13a**–**e** in high yields. The structures of the triazoles were established from the corresponding ^1^H- and ^13^C-NMR spectra.

The cell growth-inhibitory activities of the triazoles and their precursors were determined *in vitro* against a panel of human adherent cervical (HeLa, SiHa and C33A), breast (MCF-7, MDA-MB-231, MDA-MB-361 and T47D) and ovarian (A2780) cell lines by means of MTT assays (Table 1). The 3-benzyl ether of the 13β-d-secoalcohol **1** selectively inhibited the growth of MCF-7 cells, with inhibition values >80% at 10 μM. Its C-13 epimer **6** at 10 μM displayed <50% inhibition on all the examined cell lines. The 3-OH derivatives **7** and **8** exerted low antiproliferative potentials at both concentrations (even at 30 μM). Among the propargyl derivatives **9** and **10**, only the 13β epimer **9** inhibited the growth of nearly all the cell lines by >50% at 30 μM. As concerns the triazoles, **12a** displayed the highest antiproliferative activity against A2780, HeLa and C33A, with inhibition values >80% at 10 μM. The *p*-methyl derivative **12b** displayed somewhat lower potential against the abovementioned cell lines. Compounds **12c** and **12d** inhibited the growth of only two cell lines (A2780 and C33A) by >80% at 30 μM. The 13α-epimeric triazole **13a** displayed substantial antiproliferative potential against A2780, Hela and C33A. **13b** inhibited the growth of nearly all cell lines by >50% at 30 μM. The other 13α-epimeric triazoles **13c**–**e** did not influence the growth of the cell lines effectively.

Compound **12a** displayed limited growth inhibition against noncancerous human fibroblast cell line (MRC-5) with values <30% even at 30 μM.

## 3. Discussion

The benzyl protecting groups of the d-secoalcohols **1**, **6** in both the 13α- and the 13β-d-secoestrone series were removed by hydrogenolysis, using Pd/C as a catalyst. The saturation of the δ-alkenyl side-chain occurred simultaneously. The resulting 3,17-diols **7** and **8** were selectively alkylated at their 3-OH functions, taking advantage of the more acidic behavior of the phenolic over the alcoholic OH groups. The propargylations led to the desired terminal alkynes **9** or **10** in high yields. The steroidal alkynes were subjected to azide-alkyne cycloadditions under the earlier published reaction conditions [20], using a catalytic amount of CuI and PPh_3_ as an accelerating ligand.

All the CuAACs furnished the triazoles **12a**–**e** or **13a**–**e** in excellent yields. The orientation of the angular methyl group and the substitution pattern of the *N*-benzyl ring did not influence the yields of the reactions, as it was expected.

From the comparison of the ^1^H-NMR spectra of the 3-benzyl ethers **1** and **6** with those of their phenolic counterparts **7** and **8**, the absence of the proton signals of the benzylic protecting group from the aromatic region, and the presence of the singlet at around 9 ppm clearly indicates the successful removal of the protecting group. In the ^1^H-NMR spectra of **9** and **10**, the introduction of the propargyl group onto the 3-O is supported by the singlet at 2.5 ppm, which relates to the terminal alkyne function, and by the singlet of double intensity (the OCH_2_ group of the ether function). In the ^13^C-NMR spectra of the triazoles **12a**–**e** and **13a**–**e**, the two OCH_2_ and the NCH_2_ carbon signals appear in the 55–70 ppm range, indicating the presence of the *N*-benzyltriazolylmethoxy moiety on C-3. There are additional quaternary carbon signals in the aromatic region of the spectra of the triazoles, belonging to the newly introduced moiety. As concerns the epimeric character of the triazoles, C-18 in the 13β epimers **12a**–**e** appears at higher chemical shift (~25 ppm) than that in the 13α counterparts **13a**–**e** (~16 ppm).

The results of the MTT assays of the 3-OH **7** and **8** or 3-ether compounds **1**, **6**, **9** and **10** revealed their substantially lower inhibitory properties than those of some triazoles (Table 1). 3-OH derivatives **7**, **8** exerted the lowest growth inhibition, thus the presence of the phenolic OH function in the d-secoestrone seems to be disadvantageous. A bulky apolar benzyl or a smaller propargyl ether protecting group on C-3 improved the antiproliferative behavior, leading to values >80% or >50% being attained at 30 μM. Introduction of a triazolylmethyl linker between the oxygen on C-3 and the benzyl protecting group seemed to be beneficial in both C-13 epimer series. As concerns the triazoles **12a**–**e** and **13a**–**e**, the 13β epimers **12a**–**e** displayed overall higher inhibitions than their 13α counterparts **13a**–**e**. In both series, the most potent derivative **12a** or **13a** was that bearing an unsubstituted *N*-benzyl group, as observed earlier in the case of the d-secooxime **3** [11]. The characterization of the mechanism of the antiproliferative action of **12a** on the three cervical cancer cell lines in under publication [23]. However, **12a** displayed unusual behavior against the panel of breast cancer cell lines, with inhibition levels <75% even at 30 μM. It can be stated that the presence of an electron-donating *p*-alkyl or an electron-withdrawing *p*-nitro substituent on the *N*-benzyl ring of the triazoles **12c**–**e** and **13c**–**e** usually proved to be detrimental for biological activity. The inhibitory effects decreased as the size of the *p*-alkyl moiety increased. It may be noted that to date there have been no reports of the 13α-d-secoestrone derivatives with high antitumor activity.

The majority of cervical carcinomas originate from high-risk human papillomavirus (HPV) infections of the epithelial layer of the cervix, including HPV-16, -18, -31 and -35 among others [24]. HeLa is known to be an HPV-18-positive cell line [25]. SiHa and C33A differ in HPV-16 status, since only SiHa is infected by it. This pathological difference may cause a difference in antiproliferative action of the compounds against these cell lines. Our test compounds did not significantly influence the proliferation of SiHa cells, except in the cases of two ethers of the 13β epimer **1** or **9**, with values of >80% at 30 μM. However, several triazoles were similarly potent against HeLa or C33A, independently of the HPV status of the cell lines.

Substantial differences in the growth-inhibitory potential of compound **1** were determined against a panel of breast cancer cell lines differing in receptor status [26]. These cell lines included T47D (expressing the estrogen, progesterone and androgen receptors), MDA-MB-361 (expressing the estrogen receptor and HER2) and a triple-negative cell line, MDA-MB-231. Compound **1** proved to be selective against cell line MCF-7. Since the other test compounds displayed similar activities against this cell line panel, the receptor status of the cells seems irrelevant, as we earlier observed for certain recently published d-homoestrones [27].

The cancer selectivity of one of the most promising compounds **12a** was tested by means of the same MTT assay, using non-cancerous human fibroblast cells MRC5. Compound **12a** elicited growth inhibition of 24.9% ± 4.9% (mean ± SEM) when applied at a final concentration of 30 µM. The reference agent cisplatin at the same concentration caused a more substantial inhibition (70.7% ± 1.3%). On the basis of these results, it could be concluded that the selected compound displays limited growth-inhibitory action against these non-cancerous cells, indicating some selective toxicity towards fast growing cancer cells.

## 4. Materials and Methods

### 4.1. General Information

Melting points (mp) were determined with a Kofler hot-stage apparatus and are uncorrected. Elemental analyses were performed with a CHN analyzer model 2400 (Perkin-Elmer, Waltham, MA, USA). Thin-layer chromatography: silica gel 60 F_254_; layer thickness 0.2 mm (Merck, New York, NY, USA); eluents: (A) 2% ethyl acetate/98% dichloromethane, (B) 10% ethyl acetate/90% dichloromethane; detection with iodine or UV (365 nm) after spraying with 5% phosphomolybdic acid in 50% aqueous phosphoric acid and heating at 100–120 °C for 10 min. Flash chromatography: silica gel 60, 40–63 μm (Merck). ^1^H-NMR spectra were recorded in CDCl_3_ solution (if not otherwise stated) with a DRX-500 instrument (Bruker, Billerica, MA, USA) at 500 MHz, with Me_4_Si as internal standard. ^13^C-NMR spectra were recorded with the same instrument at 125 MHz under the same conditions.

### 4.2. Chemistry

#### 4.2.1. General Procedure for the Synthesis of 3-Benzyloxy-d-secoalcohols **1**, **6**

d-secoaldehyde **4** or **5** [10] (374 mg, 1.00 mmol) was dissolved in a 1:1 mixture of dichloromethane and methanol (10 mL) in an ice-water bath and potassium borohydride (270 mg, 5.00 mmol) was added in small portions. The mixture was allowed to stand at room temperature for 0.5 h, then diluted with water and extracted with dichloromethane. The combined organic phases were washed with water until neutral and dried over sodium sulfate. The crude product was subjected to flash chromatography with dichloromethane as eluent.

*3-Benzyloxy-13α-hydroxymethyl-14β-(prop-2-enyl)-des-d-estra-1,3,5(10)-triene* (**1**). As described in Section 4.2.1, d-secoaldehyde **4** (374 mg, 1.00 mmol) was reacted with potassium borohydride (270 mg, 5.00 mmol). Compound **1** is identical with the compound described in the literature [10]: oil, R_f_ = 0.53 (A). ^1^H-NMR δ ppm 0.80 (s, 3H, 18-H_3_), 2.85 (m, 2H, 6-H_2_), 3.30 and 3.61 (2× m, 2× 1H, 17-H_2_), 5.03 (m, 2H, 16a-H_2_), 5.04 (s, 2H, OCH_2_), 5.93 (m, 1H, 16-H), 6.73 (d, 1H, *J* = 2.3 Hz, 4-H), 6.79 (dd, 1H, *J* = 8.5 Hz, *J* = 2.3 Hz, 2-H), 7.22 (d, 1H, *J* = 8.5 Hz, 1-H), 7.32 (t, 1H, *J* = 7.3 Hz, 4′-H), 7.38 (t, 2H, *J* = 7.3 Hz, 3′-H and 5′-H), 7.43 (d, 2H, *J* = 7.3 Hz, 2′-H and 6′-H).

*3-Benzyloxy-13β-hydroxymethyl-14β-(prop-2-enyl)-des-d-estra-1,3,5(10)-triene* (**6**). As described in Section 4.2.1, d-secoaldehyde **5** (374 mg, 1.00 mmol) was reacted with potassium borohydride (270 mg, 5.00 mmol). Compound **6** was obtained as a white solid. Yield: 347 mg (92%). Mp 50–52 °C, R_f_ = 0.47 (A). Anal. Calcd. for C_26_H_32_O_2_: C, 82.94; H, 8.57. Found: C, 83.05; H, 8.66. ^1^H-NMR δ ppm 1.06 (s, 3H, 18-H_3_); 2.82 (m, 2H, 6-H_2_); 3.53 and 3.72 (2× d, 2× 1H, *J* = 10.8 Hz, 17-H_2_); 4.96–5.07 (overlapping multiplets, 4H, 16a-H_2_, OCH_2_); 5.87 (m, 1H, 16-H); 6.71 (s, 1H, 4-H); 6.79 (d, 1H, *J* = 8.3 Hz, 2-H); 7.21 (d, 1H, *J* = 8.3 Hz, 1-H); 7.32 (t, 1H, *J* = 6.9 Hz, 4′-H); 7.38 (t, 2H, *J* = 7.1 Hz, 3′-H and 5′-H); 7.42 (d, 2H, *J* = 6.7 Hz, 2′-H and 6′-H), ^13^C-NMR δ ppm 25.3 (C-18); 26.5; 27.8; 30.3; 32.4; 35.6; 38.8; 41.2; 43.7; 50.7; 64.5 (C-17); 69.9 (OCH_2_); 112.4 (C-2); 114.5 (C-4); 114.6 (C-16a); 126.3 (C-1); 127.4 (2C:C-3′,5′); 127.8; 128.5 (2C:C-2′,6′); 133.0 (C-4′); 137.3 (C-10); 137.9 (C-5); 140.2 (C-16); 156.8 (C-3).

#### 4.2.2. General Procedure for the Synthesis of 3-Hydroxy-d-secoestrones **7**, **8**

A suspension of **1** or **6** (376 mg, 1.00 mmol) and Pd/C (0.30 g, 10%) in ethyl acetate (20 mL) was subjected to 20 bar of hydrogen pressure at room temperature for 3 h. The catalyst was then removed by filtration through a short pad of silica gel. After evaporation of the solvent *in vacuo*, the crude product was subjected to flash chromatography with dichloromethane as eluent.

*3-Hydroxy-13α-hydroxymethyl-14β-propyl-des-d-estra-1,3,5(10)-triene* (**7**). As described in Section 4.2.2, compound **1** (376 mg, 1.00 mmol) was subjected to hydrogenolysis. Compound **7** is identical with compound described in the literature [10]: Mp 60–62 °C, R_f_ = 0.17 (A). ^1^H-NMR δ ppm 0.77 (s, 3H, 18-H_3_), 2.82 (m, 2H, 6-H_2_), 3.35 and 3.52 (2× d, 2× 1H, *J* = 10.9 Hz, 17-H_2_), 6.56 (d, 1H, *J* = 2.3 Hz, 4-H), 6.63 (dd, 1H, *J* = 8.5 Hz, *J* = 2.3 Hz, 2-H), 7.16 (d, 1H, *J* = 8.5 Hz, 1-H).

*3-Hydroxy-13β-hydroxymethyl-14β-propyl-des-d-estra-1,3,5(10)-triene* (**8**). As described in Section 4.2.2, compound **6** (376 mg, 1.00 mmol) was subjected to hydrogenolysis. The chromatographic purification of the crude product yielded **8** as a white solid (268 mg, 93%). Mp 50‒52 °C, R_f_ = 0.27 (A). Anal. Calcd. for C_19_H_28_O_2_: C, 79.12; H, 9.78. Found: C, 79.25; H, 9.96. DMSO-*d*_6_^1^H-NMR δ ppm 0.89 (t, 3H, *J* = 6.7 Hz, 16a-H_3_); 1.17 (s, 3H, 18-H_3_); 2.69 (m, 2H, 6-H_2_); 3.19 and 3.48 (2× m, 2× 1H, 17-H_2_); 4.18 (s, 1H, 17-OH); 6.41 (s, 1H, 4-H); 6.50 (dd, 1H, *J* = 1.76 Hz, *J* = 8.2 Hz, 2-H); 7.04 (d, 1H, *J* = 8.5 Hz, 1-H); 8.96 (s, 1H, 3-OH), ^13^C-NMR δ ppm 14.4 (C-16a); 25.0; 25.2 (C-18); 26.3; 27.4; 29.8; 30.3; 35.1; 38.0; 41.3; 43.2; 50.8; 61.5 (C-17); 112.7 (C-2); 114.5 (C-4); 126.0 (C-1); 130.6 (C-10); 136.9 (C-5); 154.8 (C-3).

#### 4.2.3. General Procedure for the Synthesis of 3-(Prop-2-inyloxy)-d-secoestrones **9**, **10**

3-Hydroxy-d-secoalcohol **7** or **8** (288 mg, 1.00 mmol) was dissolved in acetone (10 mL), propargyl bromide (0.17 mL (80 wt % in toluene), 1.50 mmol) and potassium carbonate (968 mg, 7.00 mmol) were added. The reaction mixture was stirred at 70 °C for 24 h, the solvent was evaporated off, and the residue was subjected to flash chromatography with 2% ethyl acetate/98% dichloromethane as eluent.

*3-(Prop-2-ynyloxy)-13α-hydroxymethyl-14β-propyl-des-d-estra-1,3,5(10)-triene* (**9**). As described in Section 4.2.3, 3-hydroxy-d-secoalcohol **7** (288 mg, 1.00 mmol) was reacted with propargyl bromide (0.17 mL (80 wt % in toluene), 1.50 mmol). Compound **9** was obtained as a white solid (280 mg, 86%). Mp 41–43 °C, R_f_ = 0.40 (A). Anal. Calcd. for C_22_H_30_O_2_: C, 80.94; H, 9.26. Found: C, 81.02; H, 9.35. ^1^H-NMR δ ppm 0.78 (s, 3H, 18-H_3_); 0.92 (t, 3H, *J* = 6.9 Hz, 16a-H_3_); 2.50 (s, 1H, C≡CH); 2.86 (m, 2H, 6-H_2_); 3.34 and 3.52 (2× d, 2× 1H, *J* = 10.9 Hz, 17-H_2_); 4.66 (s, 2H, OCH_2_); 6.70 (d, 1H, *J* = 2.3 Hz, 4-H); 6.79 (dd, 1H, *J* = 8.5 Hz, *J* = 2.3 Hz, 2-H); 7.24 (d, 1H, *J* = 8.5 Hz, 1-H), ^13^C-NMR δ ppm 14.7 (C-16a); 15.9 (C-18); 25.0; 26.4; 27.5; 30.7; 31.2; 35.6; 38.7; 41.7; 43.5; 45.2; 55.7 and 71.3 (2× OCH_2_); 74.9 (C≡CH); 78.5 (C≡CH); 112.4 (C-2); 114.5 (C-4); 126.6 (C-1); 133.8 (C-10); 138.1 (C-5); 155.4 (C-3).

*3-(Prop-2-ynyloxy)-13β-hydroxymethyl-14β-propyl-des-d-estra-1,3,5(10)-triene* (**10**). As described in Section 4.2.3, 3-hydroxy-d-secoalcohol **8** (288 mg, 1.00 mmol) was reacted with propargylbromide (0.17 mL (80 wt % in toluene), 1.50 mmol). Compound **10** was obtained as a white solid (271 mg, 83%). Mp 41‒43 °C, R_f_ = 0.50 (A). Anal. Calcd. for C_22_H_30_O_2_: C, 80.94; H, 9.26. Found: C, 80.87; H, 9.42. ^1^H-NMR δ ppm 0.92 (t, 3H, *J* = 6.9 Hz, 16a-H_3_); 1.03 (s, 3H, 18-H_3_); 2.50 (s, 1H, C≡CH); 2.84 (m, 2H, 6-H_2_); 3.47 and 3.73 (2× d, 2× 1H, *J* = 10.9 Hz, 17-H_2_); 4.65 (s, 2H, OCH_2_); 6.68(d, 1H, *J* = 2.3 Hz, 4-H); 6.78 (dd, 1H, *J* = 8.5 Hz, *J* = 2.3 Hz, 2-H); 7.22 (d, 1H, *J* = 8.5 Hz, 1-H), ^13^C-NMR δ ppm 14.6 (C-16a); 24.9 (C-18); 25.5; 26.6; 27.7; 30.6; 31.0; 35.2; 38.6; 41.7; 43.7; 51.4; 55.7 and 64.1 (2× OCH_2_); 75.2 (C≡CH); 78.9 (C≡CH); 112.4 (C-2); 114.5 (C-4); 126.5 (C-1); 133.8 (C-10); 137.9 (C-5); 155.4 (C-3).

#### 4.2.4. General Procedure for the “Click” Reaction

To a stirred solution of 3-(prop-2-inlyoxy)-d-secoalcohol **9** or **10** (326 mg, 1.00 mmol) in toluene (5 mL), PPh_3_ (52 mg, 0.20 mmol), CuI (19 mg, 0.10 mmol), DIPEA (0.52 ml, 3.00 mmol) and the appropriate benzyl azide **11** (1 equiv., see [28,29,30,31] for their preparation) were added. The reaction mixture was refluxed for 2 h, allowed to cool and evaporated *in vacuo*. The residue **12a**–**e**, **13a**–**e** was purified by flash chromatography with 10% ethyl acetate/90% dichloromethane as eluent.

*3-[{1-Benzyl-1H-1,2,3-triazol-4-yl}methoxy]-13α-hydroxymethyl-14β-propyl-des-d-estra-1,3,5(10)-triene* (**12a**). As described in Section 4.2.4, alkyne **9** (326 mg, 1.00 mmol) was reacted with benzyl azide **11a** (133 mg, 1.0 mmol). Yield: 428 mg (93%). Mp 41‒43 °C, R_f_ = 0.46 (B). Anal. Calcd. for C_29_H_37_N_3_O_2_: C, 75.78; H, 8.11. Found: C, 75.94; H, 8.25. ^1^H-NMR δ ppm 0.77 (s, 3H, 18-H_3_); 0.92 (t, 3H, *J* = 6.9 Hz, 16a-H_3_); 2.84 (m, 2H, 6-H_2_); 3.34 and 3.52 (2× d, 2× 1H, *J* = 10.9 Hz, 17-H_2_); 5.16 (s, 2H, OCH_2_); 5.53 (s, 2H, NCH_2_); 6.69 (d, 1H, *J* = 2.3 Hz, 4-H); 6.77 (dd, 1H, *J* = 8.5 Hz, *J* = 2.3 Hz, 2-H); 7.21 (d, 1H, *J* = 8.5 Hz, 1-H); 7.28 (dd, 2H, *J* = 8.6 Hz, *J* = 2.9 Hz, 2′-H and 6′-H), 7.38 (overlapping multiplets, 3H, 3′-H, 4′-H and 5′-H); 7.52 (s, 1H, C=CH), ^13^C-NMR δ ppm 14.6 (C-18); 16.0 (C-16a); 25.0; 26.4; 27.4; 30.6; 31.2; 35.6; 38.7; 41.7; 43.5; 45.2; 54.2 (NCH_2_); 62.1 (OCH_2_); 71.3 (C-17); 112.4 (C-2); 114.4 (C-4); 122.5 (C=CH); 126.6 (C-1); 128.1 (2C: C-3′,5′); 128.8 (C-4′); 129.1 (2C: C-2′,6′); 133.5 (C-10); 134.4 (C-1′); 138.1 (C-5); 144.9 (C=CH); 156.0 (C-3).

*3-[{1-(4-Methylbenzyl)-1H-1,2,3-triazol-4-yl}methoxy]-13α-hydroxymethyl-14β-propyl-des-d-estra-1,3,5 (10)-triene* (**12b**). As described in Section 4.2.4, alkyne **9** (326 mg, 1.00 mmol) was reacted with 4-methylbenzyl azide **11b** (147 mg, 1.00 mmol). Yield: 436 mg (92%). Mp 50‒52 °C, R_f_ = 0.26 (B). Anal. Calcd. for C_30_H_39_N_3_O_2_: C, 76.07; H, 8.30. Found: C, 75.92; H, 8.54. ^1^H-NMR δ ppm 0.77 (s, 3H, 18-H_3_); 0.91 (t, 3H, *J* = 6.9 Hz, 16a-H_3_); 2.35 (s, 3H, tolyl-CH_3_); 2.83 (m, 2H, 6-H_2_); 3.34 and 3.52 (2× d, 2× 1H, *J* = 10.9 Hz, 17-H_2_); 5.15 (s, 2H, OCH_2_); 5.48 (s, 2H, NCH_2_); 6.68 (d, 1H, *J* = 2.3 Hz, 4-H); 6.76 (dd, 1H, *J* = 8.5 Hz, *J* = 2.3 Hz, 2-H); 7.17‒7.20 (overlapping multiplets, 6H, 1-H, C=CH, 2′-H, 3′-H, 5′-H and 6′-H), ^13^C-NMR δ ppm 14.6 (C-18); 15.9 (C-16a); 21.1 (tolyl-CH_3_); 24.9; 26.4; 27.4; 30.6; 31.2; 35.6; 38.7; 41.7; 43.5; 45.3; 54.2 (NCH_2_); 62.1 (OCH_2_); 71.3 (C-17); 112.4 (C-2); 114.4 (C-4); 122.4 (C=CH); 126.5 (C-1); 128.2 (2C: C-3′,5′); 129.7 (2C: C-2′,6′); 131.3 and 133.5 (C-10 and C-4′); 138.1 (C-5); 138.7 (C-1′); 144.7 (C=CH); 156.0 (C-3).

*3-[{1-(4-[Prop-2-yl]benzyl)-1H-1,2,3-triazol-4-yl}methoxy]-13α-hydroxymethyl-14β-propyl-des-d-estra-1,3,5(10)-triene* (**12c**). As described in Section 4.2.4, alkyne **9** (326 mg, 1.00 mmol) was reacted with 4-(prop-2-yl)-benzyl azide **11c** (175 mg, 1.00 mmol). Yield: 452 mg (90%). Mp 41‒43 °C, R_f_ = 0.30 (B). Anal. Calcd. for C_32_H_43_N_3_O_2_: C, 76.61; H, 8.64. Found: C, 76.85; H, 8.76. ^1^H-NMR δ ppm 0.77 (s, 3H, 18-H_3_); 0.91 (t, 3H, *J* = 6.9 Hz, 16a-H_3_); 1.24 (d, 6H, 2× prop-2-yl-CH_3_); 2.83 (m, 2H, 6-H_2_); 3.33 and 3.52 (2× d, 2× 1H, *J* = 10.9 Hz, 17-H_2_); 5.19 (s, 2H, OCH_2_); 5.50 (s, 2H, NCH_2_); 6.68 (d, 1H, *J* = 2.3 Hz, 4-H); 6.75 (dd, 1H, *J* = 8.5 Hz, *J* = 2.3 Hz, 2-H); 7.22‒7.24 (overlapping multiplets, 5H, 1-H, 2′-H, 3′-H, 5′-H and 6′-H); 7.55 (s, 1H, C=CH), ^13^C-NMR δ ppm 14.6 and 15.9 (C-18 and C-16a); 23.8 (2C: CH(CH_3_)_2_); 24.9; 26.4; 27.4; 30.6; 31.2; 33.8 (CH(CH_3_)_2_); 35.6; 38.7; 41.7; 43.5; 45.2; 54.0 (NCH_2_); 62.0 (OCH_2_); 71.2 (C-17); 112.3 (C-2); 114.3 (C-4); 122.5 (C=CH); 126.5 (C-1); 127.1 (2C: C-3′,5′); 128.2 (2C: C-2′,6′); 131.7 (C-1′); 133.5 (C-10); 138.1 (C-5); 144.7 (C-4′); 149.6 (C=CH); 156.1 (C-3).

*3-[{1-(4-tert-Butylbenzyl)-1H-1,2,3-triazol-4-yl}methoxy]-13α-hydroxymethyl-14β-propyl-des-d-estra-1,3,5 (10)-triene* (**12d**). As described in Section 4.2.4, alkyne **9** (326 mg, 1.00 mmol) was reacted with 4-*tert*-butylbenzyl azide **11d** (189 mg, 1.00 mmol). Yield: 475 mg (92%). Mp 58‒60 °C, R_f_ = 0.32 (B). Anal. Calcd. for C_33_H_45_N_3_O_2_: C, 76.85; H, 8.79. Found: C, 75.98; H, 8.95. ^1^H-NMR δ ppm 0.77 (s, 3H, 18-H_3_); 0.91 (t, 3H, *J* = 6.9 Hz, 16a-H_3_); 1.31 (s, 9H, 3× ^t^Bu-CH_3_); 2.83 (m, 2H, 6-H_2_); 3.33 and 3.52 (2× d, 2× 1H, *J* = 10.9 Hz, 17-H_2_); 5.18 (s, 2H, OCH_2_); 5.50 (s, 2H, NCH_2_); 6.68 (d, 1H, *J* = 2.3 Hz, 4-H); 6.77 (dd, 1H, *J* = 8.5 Hz, *J* = 2.3 Hz, 2-H); 7.20‒7.24 (overlapping multiplets, 3H, 1-H, 2′-H and 6′-H); 7.39 (d, 2H, *J* = 8.1 Hz, 3′-H and 5′-H); 7.54 (s, 1H, C=CH), ^13^C-NMR δ ppm 14.7 and 15.9 (C-18 and C-16a); 24.9; 26.4; 27.4; 30.7; 31.1 (3C: C(CH_3_)_3_); 31.2; 34.6 (C(CH_3_)_3_); 35.6; 38.7; 41.7; 43.5; 45.2; 54.0 (NCH_2_); 62.1 (OCH_2_); 71.3 (C-17); 112.4 (C-2); 114.4 (C-4); 122.5 (C=CH); 126.0 (2C: C-3′,5′); 126.6 (C-1); 127.9 (2C: C-2′,6′); 131.3 (C-1′); 133.5 (C-10); 138.1 (C-5); 144.8 (C-4′); 151.9 (C=CH); 156.0 (C-3).

*3-[{1-(4-Nitrobenzyl)-1H-1,2,3-triazol-4-yl}methoxy]-13α-hydroxymethyl-14β-propyl-des-d-estra-1,3,5(10)-triene* (**12e**). As described in Section 4.2.4, alkyne **9** (326 mg, 1.00 mmol) was reacted with 4-nitrobenzyl azide **11e** (178 mg, 1.00 mmol). Yield: 475 mg, 94%). Mp 65‒67 °C, R_f_ = 0.20 (B). Anal. Calcd. for C_29_H_36_N_4_O_4_: C, 69.02; H, 7.19. Found: C, 69.15; H, 7.02. ^1^H-NMR δ ppm 0.77 (s, 3H, 18-H_3_); 0.91 (t, 3H, *J* = 6.9 Hz, 16a-H_3_); 2.83 (m, 2H, 6-H_2_); 3.34 and 3.52 (2× d, 2× 1H, *J* = 10.9 Hz, 17-H_2_); 5.19 (s, 2H, OCH_2_); 5.64 (s, 2H, NCH_2_); 6.67 (d, 1H, *J* = 2.3 Hz, 4-H); 6.76 (dd, 1H, *J* = 8.5 Hz, *J* = 2.3 Hz, 2-H); 7.21 (d, 1H, *J* = 8.5 Hz, 1-H); 7.40 (d, 2H, *J* = 8.6 Hz, 2′-H, 6′-H); 7.62 (s, 1H, C=CH); 8.22 (d, *J* = 8.6 Hz, 2H, 3′-H, 5′-H), ^13^C-NMR δ ppm 14.6 (C-18); 15.9 (C-16a); 24.9; 26.4; 27.4; 30.7; 31.2; 35.6; 38.7; 41.7; 43.5; 45.2; 53.1 (NCH_2_); 62.0 (OCH_2_); 71.2 (C-17); 112.3 (C-2); 114.3 (C-4); 122.8 (CH=C); 124.3 (2C: C-3′,5′); 126.6 (C-1); 128.6 (2C: C-2′,6′); 133.7 (C-10); 138.2 (C-5); 141.5 and 145.5 (C-1′ and C=CH); 148.1 (C-4′); 155.9 (C-3).

*3-[{1-Benzyl-1H-1,2,3-triazol-4-yl}methoxy]-13β-hydroxymethyl-14β-propyl-des-d-estra-1,3,5(10)-triene* (**13a**). As described in Section 4.2.4, alkyne **10** (326 mg, 1.00 mmol) was reacted with benzyl azide **11a** (133 mg, 1.00 mmol). Yield: 437 mg (95%). Oil, R_f_ = 0.19 (B). Anal. Calcd. for C_29_H_37_N_3_O_2_: C, 75.78; H, 8.11. Found: C, 75.93; H, 8.02. ^1^H-NMR δ ppm 0.89 (t, 3H, *J* = 7.2 Hz, 16a-H_3_); 1.00 (s, 3H, 18-H_3_); 2.80 (m, 2H, 6-H_2_); 3.44 and 3.70 (2× d, 2× 1H, *J* = 10.9 Hz, 17-H_2_); 5.13 (s, 2H, OCH_2_); 5.50 (s, 2H, NCH_2_); 6.66 (d, 1H, *J* = 2.4 Hz, 4-H); 6.73 (dd, 1H, *J* = 8.5 Hz, *J* = 2.1 Hz, 2-H); 7.16 (d, 1H, *J* = 8.5 Hz, 1-H); 7.24 (d, 2H, *J* = 7.6 Hz, 2′-H and 6′-H), 7.35 (overlapping multiplets, 3H, 3′-H, 4′-H and 5′-H); 7.51 (s, 1H, C=CH), ^13^C-NMR δ ppm 14.5 (C-16a); 24.9 (C-18); 25.5; 26.6; 27.7; 30.5; 30.9; 35.2; 38.5; 41.6; 43.7; 51.4; 54.2 (NCH_2_); 62.0 and 64.0 (OCH_2_ and C-17); 112.3 (C-2); 114.3 (C-4); 122.5 (C=CH); 126.5 (C-1); 128.1 (2C: C-3′,5′); 128.8 (C-4′); 129.1 (2C: C-2′,6′); 133.4 (C-10); 134.4 (C-1′); 137.9 (C-5); 145.0 (C=CH); 156.0 (C-3).

*3-[{1-(4-Methylbenzyl)-1H-1,2,3-triazol-4-yl}methoxy]-13β-hydroxymethyl-14β-propyl-des-d-estra-1,3,5 (10)-triene* (**13b**). As described in Section 4.2.4, alkyne **10** (326 mg, 1.00 mmol) was reacted with 4-methylbenzyl azide **11b** (147 mg, 1.00 mmol). Yield: 431 mg (91%). Mp 49‒51 °C, R_f_ = 0.16 (B). Anal. Calcd. for C_30_H_39_N_3_O_2_: C, 76.07; H, 8.30. Found: C, 75.94; H, 8.22. ^1^H-NMR δ ppm 0.90 (t, 3H, *J* = 7.5 Hz, 16a-H_3_); 1.02 (s, 3H, 18-H_3_); 2.36 (s, 3H, tolyl-CH_3_); 2.82 (m, 2H, 6-H_2_); 3.47 and 3.73 (2× d, 2× 1H, *J* = 10.9 Hz, 17-H_2_); 5.14 (s, 2H, OCH_2_); 5.48 (s, 2H, NCH_2_); 6.68 (s, 1H, 4-H); 6.76 (d, 1H, *J* = 7.8 Hz, 2-H); 7.17 (overlapping multiplets, 6H, 1-H, C=CH, 2′,3′,5′,6′-H), ^13^C-NMR δ ppm 14.6 (C-16a); 21.1 (tolyl-CH_3_); 24.9 (C-18); 25.5; 26.6; 27.7; 30.5; 30.9; 35.2; 38.6; 41.7; 43.7; 51.4; 54.2 (NCH_2_); 62.0 and 64.1 (OCH_2_ and C-17); 112.4 (C-2); 114.3 (C-4); 122.4 (C=CH); 126.5 (C-1); 128.2 (2C: C-3′,5′); 129.8 (2C: C-2′,6′); 131.3 (C-4′); 133.4 (C-10); 137.9 (C-5); 138.8 (C-1′); 144.7 (C=CH); 156.0 (C-3).

*3-[{1-(4-[Prop-2-yl]benzyl)-1H-1,2,3-triazol-4-yl}methoxy]-13β-hydroxymethyl-14β-propyl-des-d-estra-1,3,5 (10)-triene* (**13c**). As described in Section 4.2.4, alkyne **10** (326 mg, 1.00 mmol) was reacted with 4-(prop-2-yl)-benzyl azide **11c** (175 mg, 1.00 mmol). Yield: 462 mg (92%). Mp 47‒49 °C, R_f_ = 0.19 (B). Anal. Calcd. for C_32_H_43_N_3_O_2_: C, 76.61; H, 8.64. Found: C, 76.85; H, 8.53. ^1^H-NMR δ ppm 0.91 (t, 3H, *J* = 6.8 Hz, 16a-H_3_); 1.02 (s, 3H, 18-H_3_); 1.24 (d, 2× 3H, *J* = 11.4 Hz, 2× prop-2-yl-CH_3_); 2.82 (m, 2H, 6-H_2_); 2.90 (m, 1H, prop-2-yl-CH); 3.47 and 3.73 (2× d, 2× 1H, *J* = 10.8 Hz, 17-H_2_); 5.15 (s, 2H, OCH_2_); 5.49 (s, 2H, NCH_2_); 6.68 (d, 1H, *J* = 2.2 Hz, 4H); 6.76 (dd, 1H, *J* = 8.6 Hz, *J* = 2.2 Hz, 2-H); 7.19‒7.23 (overlapping multiplets, 5H, 1-H, 2′,3′,5′,6′-H); 7.57 (s, 1H, C=CH), ^13^C-NMR δ ppm 14.5 (C-16a); 23.8 (2C: CH(CH_3_)_2_); 24.8 (C-18); 25.5; 26.6; 27.7; 30.6; 31.0; 33.8 (CH(CH_3_)_2_); 35.2; 38.6; 41.7; 43.7; 51.4; 54.2 (NCH_2_); 62.1 (OCH_2_); 64.1 (C-17); 112.4 (C-2); 114.4 (C-4); 122.6 (C=CH); 126.5 (C-1); 127.2 (2C: C-3′,5′); 128.2 (2C: C-2′,6′); 131.7 (C-1′); 133.4 (C-10); 137.9 (C-5); 149.7 (C-4′); 150.1 (C=CH); 156.0 (C-3).

*3-[{1-(4-tert-Butylbenzyl)-1H-1,2,3-triazol-4-yl}methoxy]-13β-hydroxymethyl-14β-propyl-des-d-estra-1,3,5 (10)-triene* (**13d**). As described in Section 4.2.4, alkyne **10** (326 mg, 1.00 mmol) was reacted with 4-*tert*-butylbenzyl azide **11d** (189 mg, 1.00 mmol). Yield: 470 mg (91%). Mp 58‒60 °C, R_f_ = 0.32 (B). Anal. Calcd. for C_33_H_45_N_3_O_2_: C, 76.85; H, 8.79. Found: C, 76.72; H, 8.90. ^1^H NMR δ ppm 0.91 (t, 3H, *J =* 7.2 Hz, 16a-H_3_); 1.02 (s, 3H, 18-H_3_); 1.32 (s, 3× 3H, 3× ^t^Bu-CH_3_); 2.82 (m, 2H, 6-H_2_); 3.47 and 3.73 (2× d, 2× 1H, *J* = 10.9 Hz, 17-H_2_); 5.16 (s, 2H, OCH_2_); 5.49 (s, 2H, NCH_2_); 6.67 (d, 1H, *J* = 2.2 Hz, 4H); 6.76 (dd, 1H, *J* = 8.6 Hz, *J* = 2.2 Hz, 2-H); 7.18 (d, 1H, *J* = 8.6 Hz, 1-H); 7.21 (d, 2H, *J* = 8.2 Hz, 2′, 6′-H); 7.39 (d, 2H, *J* = 8.2 Hz, 3′, 5′-H); 7.54 (s, 1H, C=CH), ^13^C-NMR δ ppm 14.5 (C-16a); 24.9 (C-18); 25.5; 26.6; 27.7; 30.5; 30.9; 31.2 (3C: C(CH_3_)_3_); 34.6 (C(CH_3_)_3_); 35.2; 38.6; 41.7; 43.7; 51.4; 54.0 (NCH_2_); 62.1 (OCH_2_); 64.1 (C-17); 112.4 (C-2); 114.3 (C-4); 122.6 (C=CH); 126.0 (2C: C-3′,5′); 126.5 (C-1); 127.9 (2C: C-2′,6′); 131.3 (C-1′); 133.4 (C-10); 137.9 (C-5); 144.7 (C-4′); 151.9 (C=CH); 156.0 (C-3).

*3-[{1-(4-Nitrobenzyl)-1H-1,2,3-triazol-4-yl}methoxy]-13β-hydroxymethyl-14β-propyl-des-d-estra-1,3,5(10)-triene* (**13e**). As described in Section 4.2.4, alkyne **10** (326 mg, 1.00 mmol) was reacted with 4-nitrobenzyl azide **11e** (178 mg, 1.00 mmol). Yield: 475 mg, 94%). Mp 50‒52 °C, R_f_ = 0.12 (B). Anal. Calcd. for C_29_H_36_N_4_O_4_: C, 69.02; H, 7.19. Found: C, 69.21; H, 7.05. ^1^H-NMR δ ppm 0.91 (t, 3H, *J* = 6.9 Hz, 16a-H_3_); 1.02 (s, 3H, 18-H_3_); 2.82 (m, 2H, 6-H_2_); 3.47 and 3.73 (2× d, 2× 1H, *J* = 10.9 Hz, 17-H_2_); 5.19 (s, 2H, OCH_2_); 5.65 (s, 2H, NCH_2_); 6.68 (s, 1H, 4-H); 6.76 (d, 1H, *J* = 8.6 Hz, 2-H); 7.20 (d, 1H, *J* = 8.6 Hz, 1-H); 7.40 (d, 2H, *J* = 8.3 Hz, 2′,6′-H); 7.64 (s, 1H, C=CH); 8.23 (d, 2H, *J* = 8.5 Hz, 3′,5′-H), ^13^C-NMR δ ppm 14.5 (C-16a); 24.9 (C-18); 25.5; 26.6; 27.7; 30.6; 31.0; 35.2; 38.6; 41.7; 43.7; 51.4; 53.3 (NCH_2_); 62.0 (OCH_2_); 64.1 (C-17); 112.3 (C-2); 114.4 (C-4); 124.3 (2C: C-3′,5′); 124.8 (C=CH); 126.6 (C-1); 128.6 (2C: C-2′,6′); 133.6 (C-10); 138.0 (C-5); 141.4 (C-1′); 144.0 (C=CH); 144.8 (C-4′); 155.9 (C-3).

### 4.3. Determination of Antiproliferative Activities

The antiproliferative properties of the prepared triazoles **12a**–**e** or **13a**–**e** and compounds **1**, **6**–**10** were determined on a panel of human adherent gynecological cancer cell lines. MCF-7, MDA-MB-231, MDA-MB-361 and T47D were isolated from breast cancers differing in biochemical background, while HeLa, SiHa and C33A cells were from cervical cancers of various pathological histories, and A2780 cells were isolated from ovarian cancer. Non-cancerous human fibroblast cells (MRC-5) was additionally used to assess the cancer selectivity of the most effective compound. All cell lines were purchased from European Collection of Cell Cultures (ECCAC, Salisbury, UK) except for SiHa and C33A, which were obtained from LGC Standards GmbH (Wesel, Germany). Cells were cultivated in minimal essential medium supplemented with 10% fetal bovine serum, 1% non-essential amino acids and an antibiotic-antimycotic mixture. All media and supplements were obtained from Lonza Group Ltd., (Basel, Switzerland). Near-confluent cancer cells were seeded onto a 96-well microplate (5000 cells/well except for C33A and MDA-MB-361, which were seeded at 10,000/well) and, after overnight standing, 200 μL new medium, containing the tested compounds at 10 and 30 µM, was added. After incubation for 72 h at 37 °C in humidified air containing 5% CO_2_, the living cells were assayed by the addition of 20 μL of 5 mg/ml 3-(4,5-dimethylthiazol-2-yl)-2,5-diphenyltetrazolium bromide (MTT) solution. MTT was converted by intact mitochondrial reductase and precipitated as purple crystals during a 4-h contact period. The medium was next removed and the precipitated formazan crystals were dissolved in 100 μL of DMSO during a 60-min period of shaking at 37 °C.

Finally, the reduced MTT was assayed at 545 nm, using a microplate reader; wells with untreated cells served as control [32]. In the case of the most active compounds (*i.e.*, higher than 70% growth inhibition at 30 µM), the assays were repeated with a set of dilutions, sigmoidal dose-response curves were fitted to the determined data and the IC_50_ values (the concentration at which the extent of cell proliferation was half that of the untreated control) were calculated by means of GraphPad Prism 4.0 (GraphPad Software, San Diego, CA, USA). All *in vitro* experiments were carried out on two microplates with at least five parallel wells. Stock solutions of the tested substances (10 mM) were prepared in DMSO. The highest DMSO content of the medium (0.3%) did not have any substantial effect on the cell proliferation. Cisplatin (Ebewe Pharma GmbH, Unterach, Austria) was used as positive control.

## 5. Conclusions

Novel antiproliferative triazolyl d-secoestrone derivatives were synthesized by introducing the triazolylmethyl linker between the 3-OH and the benzyl or *p*-substituted benzyl protecting group. The “clicking” of benzyl azides to the 3-propargyl-d-secoestrones led to potent antiproliferative compounds. The synthesized derivatives differed at two sites of the molecules: in the orientation of the angular methyl function and in the nature of the substituent present on the 3-OH group. It can be stated that both variables substantially influenced the antiproliferative behavior. The 3-OH derivatives displayed the lowest growth-inhibitory action. Etherification of the phenolic OH group improved the cytostatic properties moderately, but the incorporation of the triazolylmethyl linker between the protecting group and the 3-O nevertheless increased the inhibitory values of the compounds substantially. 13β-Methyl derivatives proved to be more potent than their 13α counterparts overall. As concerns the *p*-substituent on the *N*-benzyl ring, neither the presence of the electron-withdrawing nor that of the electron-donating group appeared to be advantageous, in contrast with the unsubstituted derivative. It can be concluded that the combination of the 13β-methyl and the 3-(*N*-benzyltriazolylmethoxy) group on the d-secoestrone scaffold intensifies the antiproliferative potential. These compounds are the most potent cell-growth-inhibitor d-secoestrones reported to date. Antiproliferative potential of 13α-d-secoestrone derivatives is a novel finding.

## Figures and Tables

**Table 1:** Antiproliferative properties of the synthesized compounds. Mean value from two independent determinations with five parallel wells; standard deviation <15%.

Comp.	Conc. (µM)	Inhibition (%) ± SEM (Calculated IC_50_) ^1^							
		A2780	Hela	SiHa	C33A	MCF-7	T47D	MDA-MB-231	MDA-MB-361
**1**	10	42.3 ± 0.9	31.4 ± 1.6	- ^2^	39.2 ± 0.6	81.3 ± 0.7	26.1 ± 2.0	-	28.2 ± 0.4
	30	97.5 ± 0.1	97.9 ± 0.3	84.3 ± 0.9	86.9 ± 0.7	97.4 ± 0.3	87.2 ± 0.9	84.5 ± 0.9	87.6 ± 0.4
						(6.4)			
**6**	10	43.6 ± 2.4	20.1 ± 1.8	-	40.4 ± 2.1	-	-	-	-
	30	55.4 ± 2.5	60.3 ± 1.4	49.7 ± 1.4	53.8 ± 1.1	36.4 ± 1.2	59.6 ± 0.8	49.3 ± 1.6	77.2 ± 1.3
**7**	10	-	-	-	40.5 ± 0.8	24.3 ± 2.6	-	-	-
	30	35.1 ± 0.8	40.7 ± 1.7	-	42.3 ± 1.8	51.6 ± 2.9	-	-	-
**8**	10	-	23.7 ± 0.9	-	-	-	-	-	-
	30	-	64.5 ± 1.1	-	-	-	23.0 ± 1.7	-	-
**9**	10	22.4 ± 1.0	21.5 ± 0.8	-	30.0 ± 1.1	-	-	36.8 ± 2.7	-
	30	70.7 ± 0.4	89.3 ± 1.9	84.5 ± 0.5	70.1 ± 0.7	52.5 ± 1.0	37.7 ± 1.3	81.0 ± 1.1	59.0 ± 2.9
**10**	10	-	-	-	-	-	-	-	-
	30	29.7 ± 1.9	36.9 ± 1.8	-	39.9 ± 1.1	23.3 ± 0.6	45.5 ± 0.6	28.2 ± 2.2	-
**12a**	10	81.5 ± 1.1	85.4 ± 0.3	21.2 ± 1.1	90.0 ± 0.3	66.3 ± 0.3	51.0 ± 1.1	53.5 ± 1.2	59.3 ± 1.4
	30	88.0 ± 0.1	91.7 ± 0.3	34.5 ± 1.2	95.1 ± 0.2	74.5 ± 1.7	54.4 ± 1.8	59.6 ± 1.8	45.2 ± 1.1
		(0.9)	(1.1) [23]		(1.8) [23]	(1.5)			
**12b**	10	96.8 ± 0.2	52.6 ± 0.9	48.1 ± 0.8	86.8 ± 0.8	71.6 ± 1.0	65.7 ± 1.4	58.3 ± 0.7	87.2 ± 0.5
	30	97.4 ± 0.1	65.4 ± 0.9	64.3 ± 1.0	93.9 ± 0.9	73.9 ± 1.0	66.4 ± 1.2	86.1 ± 0.3	89.3 ± 1.1
		(3.8)			(5.0)	(5.0)		(8.3)	(4.4)
**12c**	10	83.3 ± 0.5	27.1 ± 1.7	-	57.5 ± 1.8	-	-	33.6 ± 0.8	47.1 ± 2.9
	30	93.4 ± 0.1	66.0 ± 2.4	35.6 ± 0.3	84.6 ± 0.9	66.1 ± 1.7	53.0 ± 1.6	48.9 ± 0.7	45.6 ± 0.6
		(5.4)			(8.3)				
**12d**	10	20.5 ± 1.2	30.9 ± 3.0	-	25.3 ± 1.5	-	-	-	-
	30	29.0 ± 2.0	45.9 ± 1.1	-	64.0 ± 1.8	29.5 ± 2.7	28.9 ± 0.9	-	45.5 ± 1.2
**12e**	10	86.4 ± 0.3	46.3 ± 2.5	25.5 ± 2.0	81.4 ± 1.9	61.1 ± 1.6	41.5 ± 2.0	49.8 ± 0.7	47.5 ± 0.7
	30	89.6 ± 0.3	72.5 ± 1.1	23.2 ± 1.2	88.9 ± 0.6	63.4 ± 0.9	49.7 ± 2.5	48.8 ± 0.9	46.7 ± 1.0
		(4.6)			(5.4)	(6.6)			
**13a**	10	73.4 ± 0.9	70.0 ± 0.7	41.8 ± 1.7	80.0 ± 0.5	39.4 ± 1.2	36.6 ± 0.7	38.2 ± 2.2	65.8 ± 1.0
	30	83.8 ± 0.8	90.9 ± 0.3	37.1 ± 1.0	93.9 ± 0.1	73.8 ± 1.1	69.1 ± 0.6	59.7 ± 1.6	48.6 ± 1.4
		(3.0)	(5.3)		(4.4)				
**13b**	10	54.3 ± 0.8	33.9 ± 1.1	-	26.6 ± 1.8	-	43.2 ± 0.3	36.7 ± 1.8	-
	30	81.0 ± 0.1	74.4 ± 0.9	49.4 ± 0.8	86.1 ± 0.6	65.6 ± 1.9	89.3 ± 0.8	56.8 ± 1.2	48.7 ± 1.0
		(9.8)							
**13c**	10	-	21.3 ± 2.3	-	-	-	-	-	-
	30	52.7 ± 1.9	40.6 ± 0.9	-	41.7 ± 1.2	20.1 ± 0.8	39.4 ± 1.7	28.1 ± 0.7	25.7 ± 2.8
**13d**	10	20.0 ± 1.9	24.4 ± 1.3	-	26.1 ± 0.9	-	-	-	-
	30	44.8 ± 1.1	36.9 ± 0.8	23.0 ± 1.2	66.3 ± 0.6	30.8 ± 1.7	44.4 ± 1.7	37.5 ± 1.6	40.3 ± 2.1
**13e**	10	-	-	-	-	-	-	-	-
	30	29.1 ± 2.3	48.3 ± 2.0	-	34.2 ± 1.3	-	22.8 ± 1.9	-	-
Cisplatin	10	83.6 ± 1.2	42.6 ± 2.3	88.6 ± 0.5	83.8 ± 0.8	66.9 ± 1.8	51.0 ± 2.0	-	67.5 ± 1.0
	30	95.0 ± 0.3	99.9 ± 0.3	90.2 ± 1.8	94.0 ± 0.6	96.8 ± 0.4	57.9 ± 1.5	71.5 ± 1.2	87.8 ± 1.1
		(1.3)	(12.4)	(7.8)	(3.7)	(5.8)	(9.8)	(19.1)	(3.7)

^1^: Mean value from two independent determinations with five parallel wells; standard deviation <15%; ^2^: Inhibition values <20% are not presented.
